# Author Correction: Oxidative rearrangement of (+)-sesamin by CYP92B14 co-generates twin dietary lignans in sesame

**DOI:** 10.1038/s41467-018-04596-9

**Published:** 2018-05-25

**Authors:** Jun Murata, Eiichiro Ono, Seigo Yoroizuka, Hiromi Toyonaga, Akira Shiraishi, Shoko Mori, Masayuki Tera, Toshiaki Azuma, Atsushi J. Nagano, Masaru Nakayasu, Masaharu Mizutani, Tatsuya Wakasugi, Masayuki P. Yamamoto, Manabu Horikawa

**Affiliations:** 1 Bioorganic Research Institute, Suntory Foundation for Life Sciences (SUNBOR), 8-1-1 Seikadai, Seika, Soraku, Kyoto 619-0284 Japan; 2 Research Institute, Suntory Global Innovation Center Ltd (SIC), 8-1-1 Seikadai, Seika, Soraku, Kyoto 619-0284 Japan; 3 Graduate School of Science and Engineering, University of Toyama, 3190 Gofuku, Toyama, 930-8555 Japan; 4 Faculty of Agriculture, Ryukoku University, 1-5 Yokotani, Seta Oe, Otsu, Shiga, 520-2914 Japan; 5 JST CREST, 4-1-8 Honcho, Kawaguchi, Saitama 332-0012 Japan; 6 Graduate School of Agricultural Science, Kobe University, Kobe, 657-8501 Japan

Correction to: *Nature Communications* 10.1038/s41467-017-02053-7, published online 18 December 2017

The original version of the Supplementary Information associated with this article inadvertently omitted oligonucleotide primer sequences from Supplementary Table 3 and Supplementary Methods describing the molecular cloning of CYP92B14, CPR1 and CYP81Q cDNA fragments.

The HTML has been updated to include a corrected version of the Supplementary Information.

