# Supplementary material for: Oxidative rearrangement of (+)-sesamin by CYP92B14 co-generates twin dietary lignans in sesame
Source: Nat Commun. 2017 Dec 18;8:2155. doi: 10.1038/s41467-017-02053-7 (PMC5735135; doi:10.1038/s41467-017-02053-7)
Supplement: Supplementary file 1 — Supplementary Information [file 41467_2017_2053_MOESM1_ESM.pdf]

Supplementary Figures:

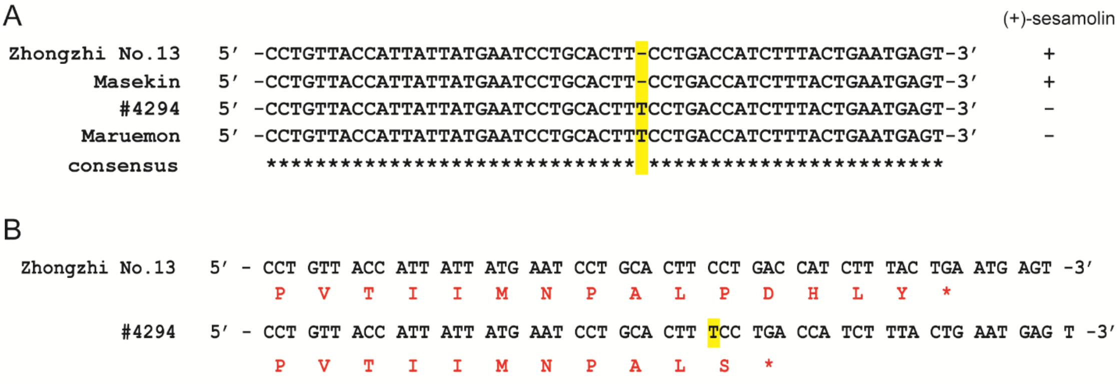

Supplementary Figure 1: SNPs among various sesame accessions in *CYP92B14*. (A) Zhongzhi No.13 and Masekin are (+)-sesamolin-accumulating accessions[1,2], whereas #4294 and Maruemon accumulate negligible amount of (+)-sesamolin[3]. Note that thymine insertion (highlighted in yellow; 1,525 bp from CYP92B14 start codon) coincides with (+)-sesamolin deficiency. Zhongzhi No. 13 is a reference accession used for genome sequencing of *S. indicum* (http://ocri-genomics.org/Sinbase/). Asterisks indicate consensus sequence. (B) Thymine insertion (highlighted in yellow) in #4294 results in a frameshift that causes an amino acid substitution (P509S) and deletion of the four C-terminal amino acids of CYP92B14. Asterisks indicate stop codons. Note that Del4C represents the putative amino acid sequence of CYP92B14 lacking C-terminal four amino acids in #4294 in this study (**Fig. 2a**).

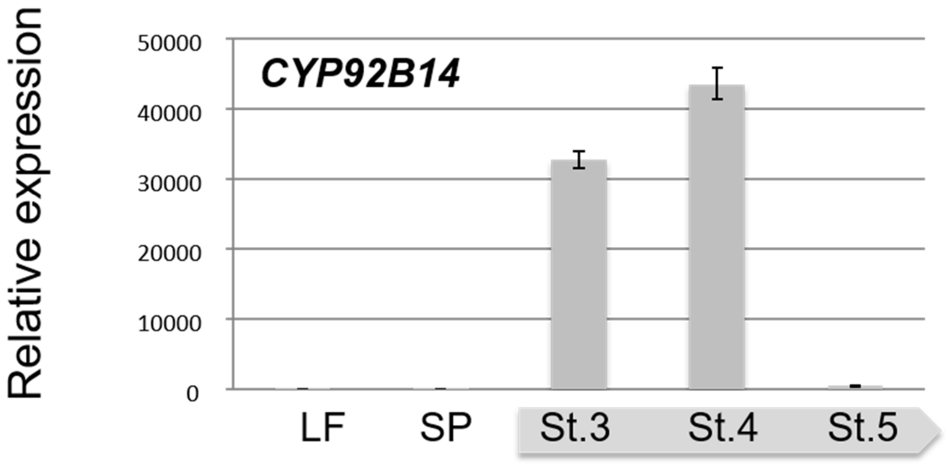

Supplementary Figure 2: qPCR analysis of *CYP92B14* expression during the course of seed development in sesame. Gene expression analysis by quantitative reverse transcription–PCR (qPCR). Transcripts of *CYP92B14* were evaluated in leaf (LF), seed pod (SP) and developing seeds at stage 3 (St.3), 4 (St.4) and fully mature 5 (St.5) by qPCR. These graphs show the relative expression of each gene in comparison with the reference gene, *Sesamum indicum* 18S rRNA. The data are presented as an expression ratio relative to the expression levels found in LF (1.00). The reaction was performed in triplicates. SD are shown by the error bars.

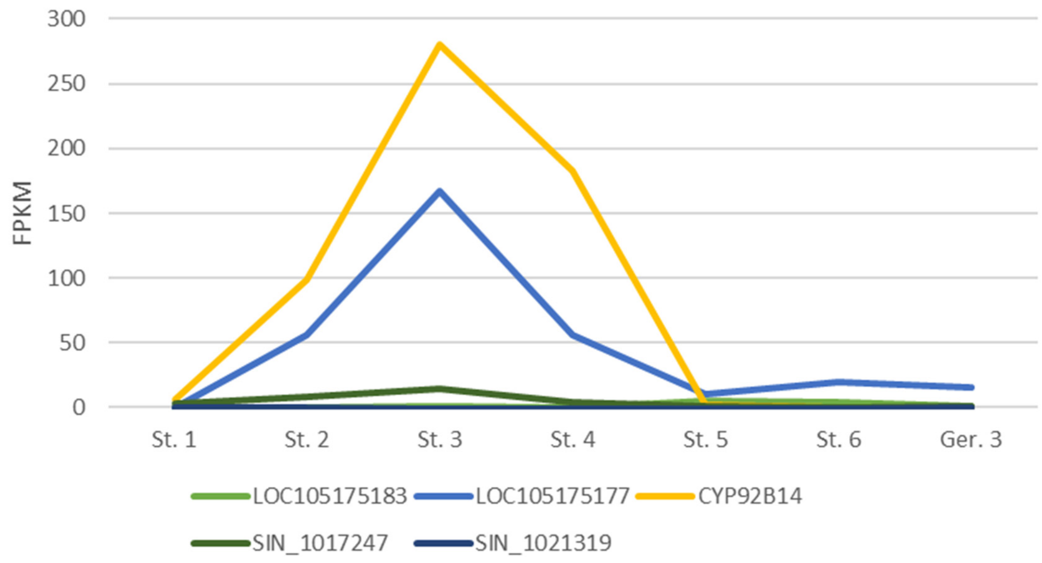

Supplementary Figure 3: RNASeq analysis of CYP92B14 and its close homologues in developing seeds of *S. indicum*. LOC105175177 was expressed to a level comparable to that of CYP92B14. In contrast, the expression levels of LOC105175183, SIN_1021319 and SIN_1017247 were at least 10 times less than compared to that of CYP92B14. FPKM: Fragments per Kilobase Megareads.

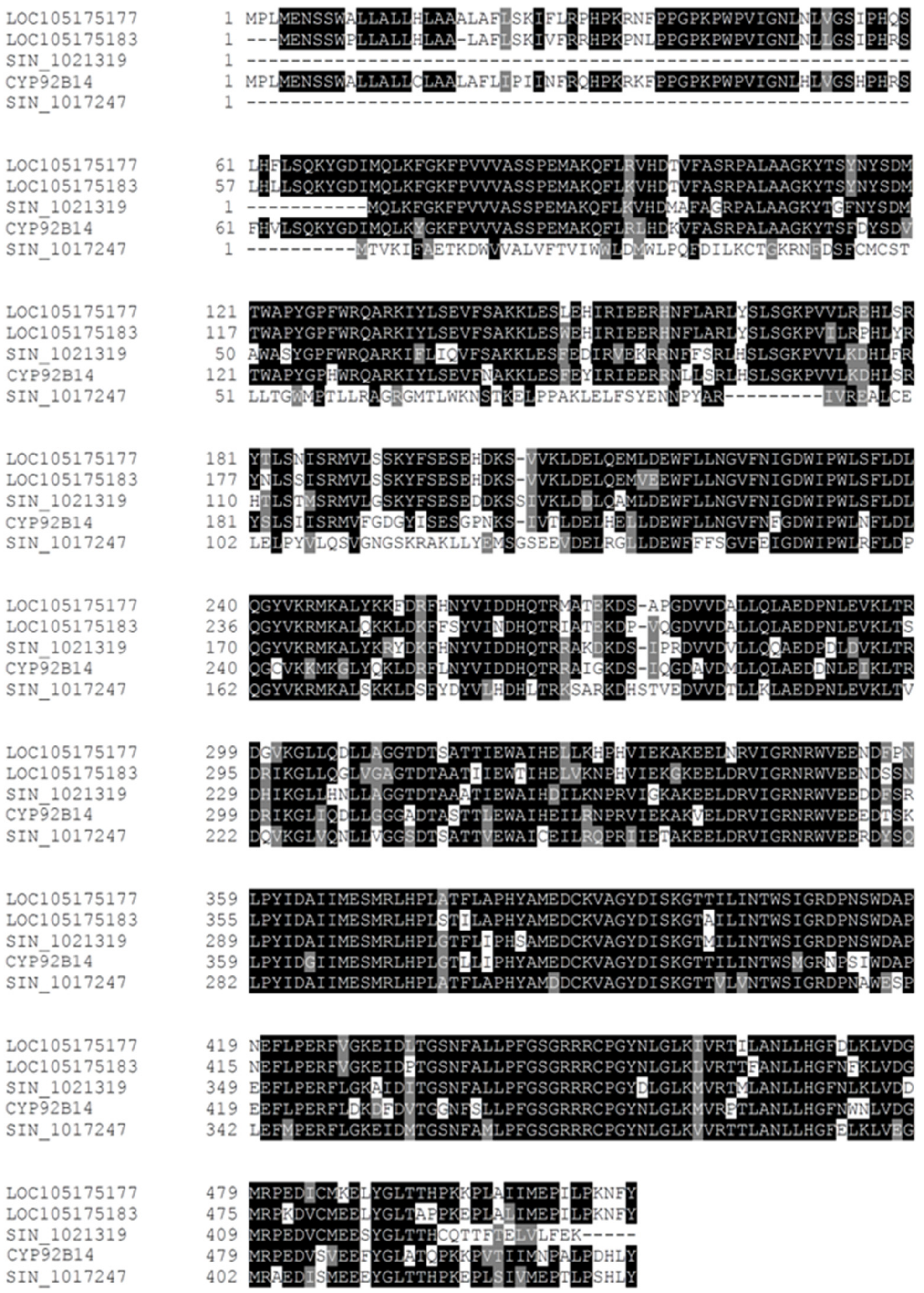

Supplementary Figure 4: Sequence alignment of CYP92B14, LOC105175177, LOC105175183, SIN_1021319 and SIN_1017247. Putative amino acid sequences of CYP92B14, LOC105175177, LOC105175183, SIN_1021319 and SIN_1017247 were aligned using CLUSTALW (http://www.genome.jp/tools-bin/clustalw) and analyzed using BoxShade (http://www.ch.embnet.org/software/BOX_form.html).

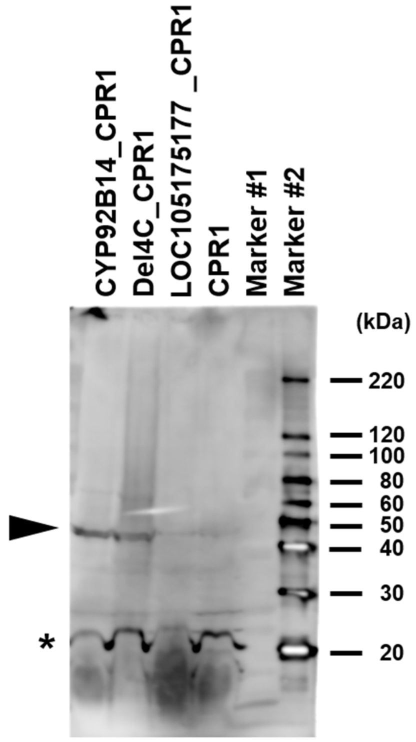

Supplementary Figure 5: Full blot of the immunoblot analysis in Figure 3c. 20 μg protein/lane of microsome fractions prepared from yeast cells expressing either CYP92B14_CPR1, Del4C_CPR1, LOC105175177_CPR1 or CPR1 were loaded onto the polyacrylamide gel and were subjected to immunoblot analysis using anti-CYP92B14 rabbit polyclonal antibodies. Anti-CYP92B14 was designed to recognize full-length CYP92B14, its deletion mutant Del4C and LOC105175177. Note that the bands that correspond to the putative size of CYP92B14 or its deletion mutant Del4C appeared in lanes CYP92B14_CPR1 and Del4C_CPR1, but not in LOC105175177. The result indicates that the expression level of LOC105175177 was not as evident as CYP92B14 or Del4C. CPR1 did not express CYP92B14 or related sesame P450 enzymes by its design. Arrowhead; CYP92B14 or Del4C, asterisk; non-specific bands. Marker #1; Prescision Plus (BioRad), Marker #2; MagicMark XP (Thermo). Secondary antibody; Rabbit IgG-HRP (GE Healthcare), ECL Select (GE Healthcare) and AI600 (GE Healthcare) was used to detect signals. Note that the apparent molecular weight (47 kDa) of CYP92B14 and Del4C is smaller than their putative molecular weight (57 kDa).

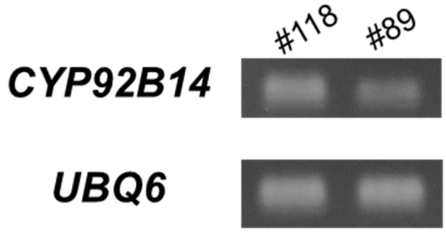

Supplementary Figure 6: RT-PCR analysis of CYP92B14 in developing seeds. Total RNA was extracted at 28 DAF (days after flowering) from developing seeds of (+)-sesamolin-accumulating (#118) and (+)–sesamolin deficient (#89) RIL plants. UBQ6 encoding the sesame ubiquitin gene was amplified as an internal control.

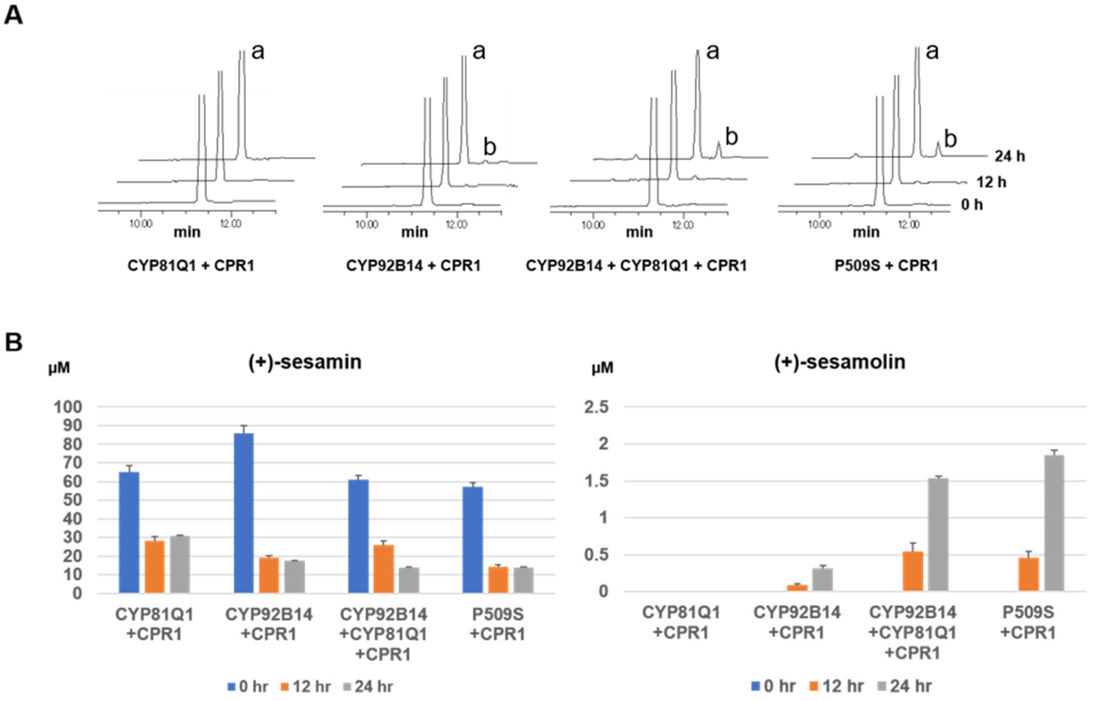

Supplementary Figure 7: The effect of CYP81Q1 co-expression and P509 amino acid substitution to (+)-sesamolin-producing activity of CYP92B14. (A) UV chromatograms of enzyme assay products obtained through yeast bioassays using yeast strains that are expressing designated genes. Detection at 283 nm. a; (+)-sesamin, b; (+)-sesamolin. 0, 12 and 24 hr after the addition of 100 µM (+)-sesamin as a substrate. (B) Quantitative measurement of consumed (+)-sesamin and produced (+)-sesamolin at various time points in the yeast bioassays. The reaction was performed in triplicates and the error bars represent SD. Note that co-expression of CYP81Q1 increased the production of (+)-sesamolin, while the effect on the consumption of (+)-sesamin was rather negligible. On the other hand, the amino acid-substitution P509S did not lead to the apparent loss of (+)-sesamolin-producing activity of CYP92B14. This is in clear contrast to the case where the activity of Del4C is completely abolished (Fig. 3). These indicate that the lack of 4 amino acids in the C-terminus, but not the replacement of proline to serine at the position 509, is responsible for the loss of CYP92B14 enzyme activity. The reaction was performed in triplicates. SD are shown by the error bars.

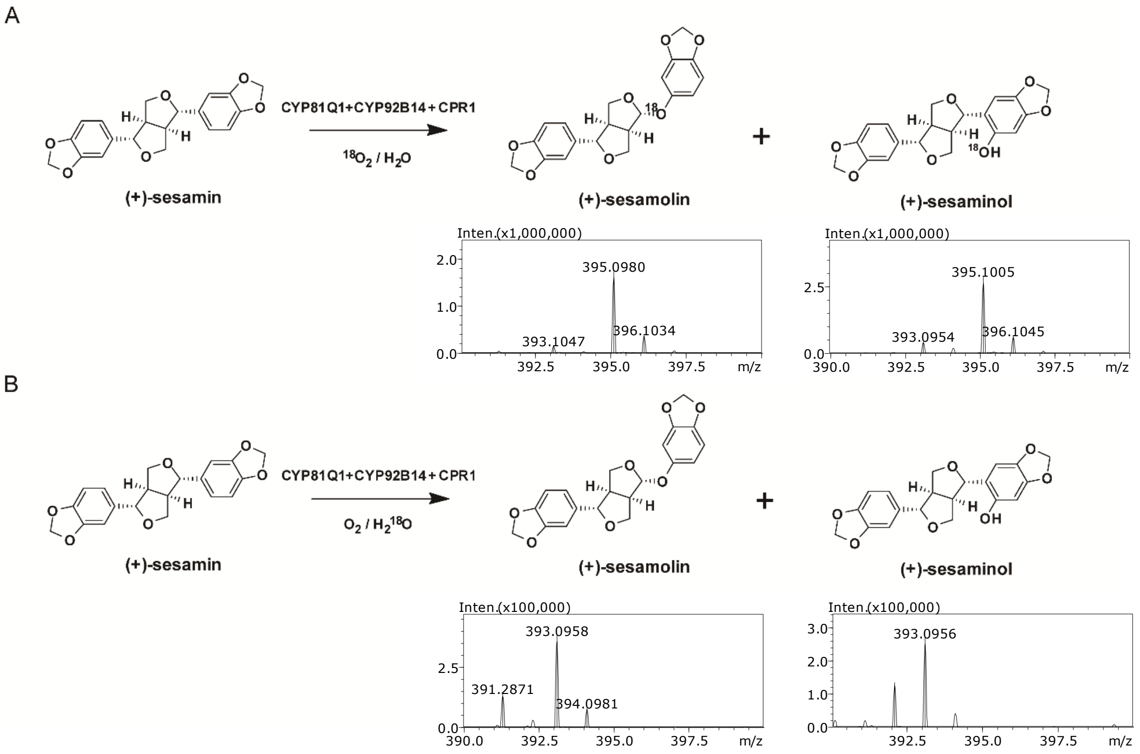

Supplementary Figure 8: Enzyme assays with ^{18}O-labeled oxygen donors reveals that CYP92B14 oxygenates (+)-sesamin using molecular oxygen. Yeast cells expressing *CYP81Q1*, *CYP92B14* and *CPR1* were subjected to bioassays using ^{18}O-labeled molecular oxygen or H_2O. (+)-Sesamin was used as a substrate. The assay products were analyzed by LC-MS. Theoretical *m/z* values of sodium adduct ions of (+)-sesamolin and (+)-sesaminol are 393.0950, while those of ^{18}O-labeled (+)-sesamolin and (+)-sesaminol are 395.0993. **(A)** The reaction scheme of the precursor ions of (+)-sesaminol and (+)-sesamolin when ^{18}O-labeled molecular oxygen was used as an oxygen donor in the bioassay. **(B)** The reaction scheme of the precursor ions of (+)-sesaminol and (+)-sesamolin when ^{18}O-labeled H_2O was used as an oxygen donor in the bioassay.

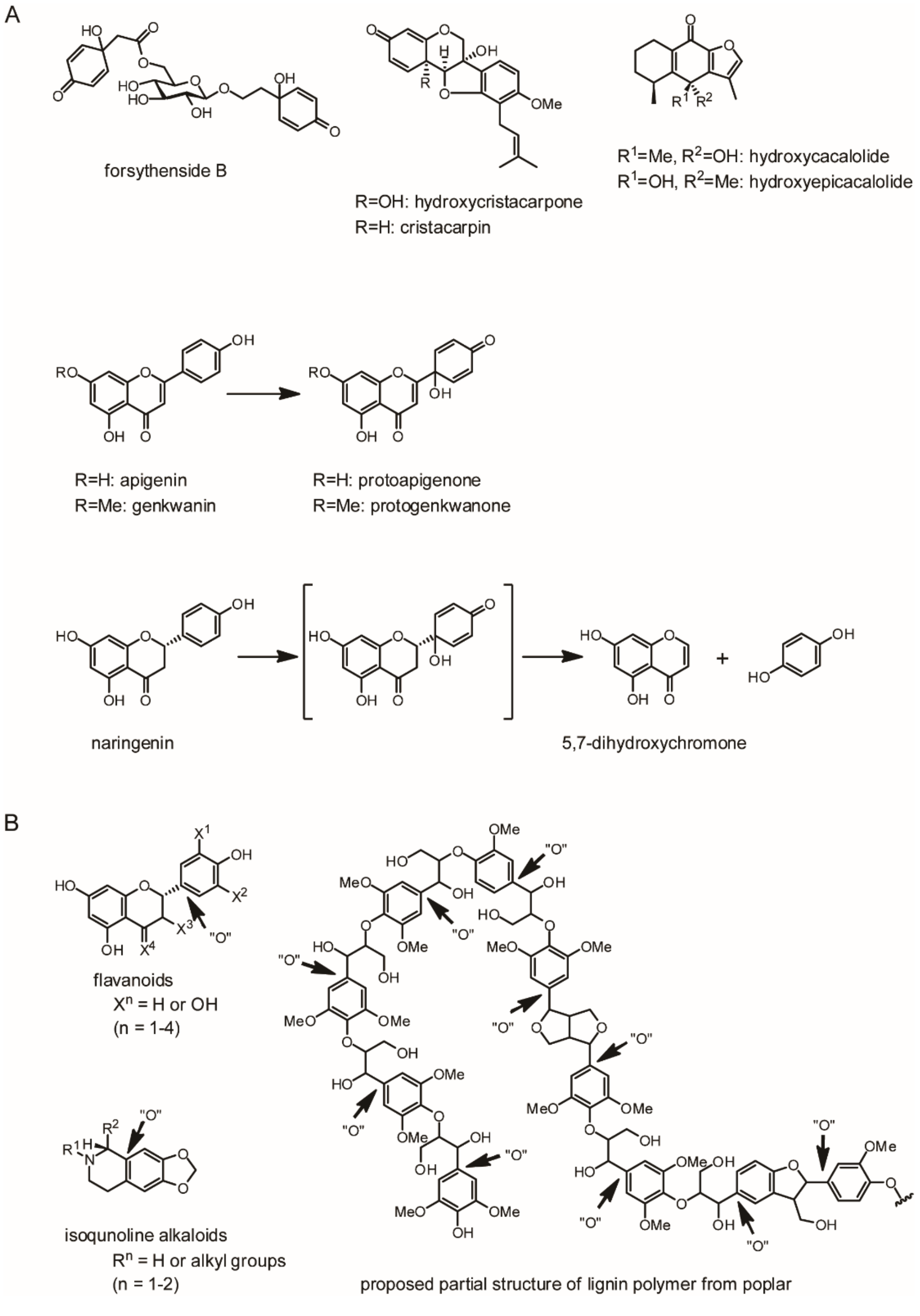

Supplementary Figure 9: Initial oxidation step of ORA is applicable to various other plant specialized metabolites. (A) Examples of specialized metabolites that are likely oxygenated through a mechanism analogous to that of the initial oxidation step of ORA[4–8]. **(B)** Additional examples of plant specialized metabolites that are likely to be subjected to ORA[9–11]. Solid arrows indicate putative sites of oxygenation by ORA.

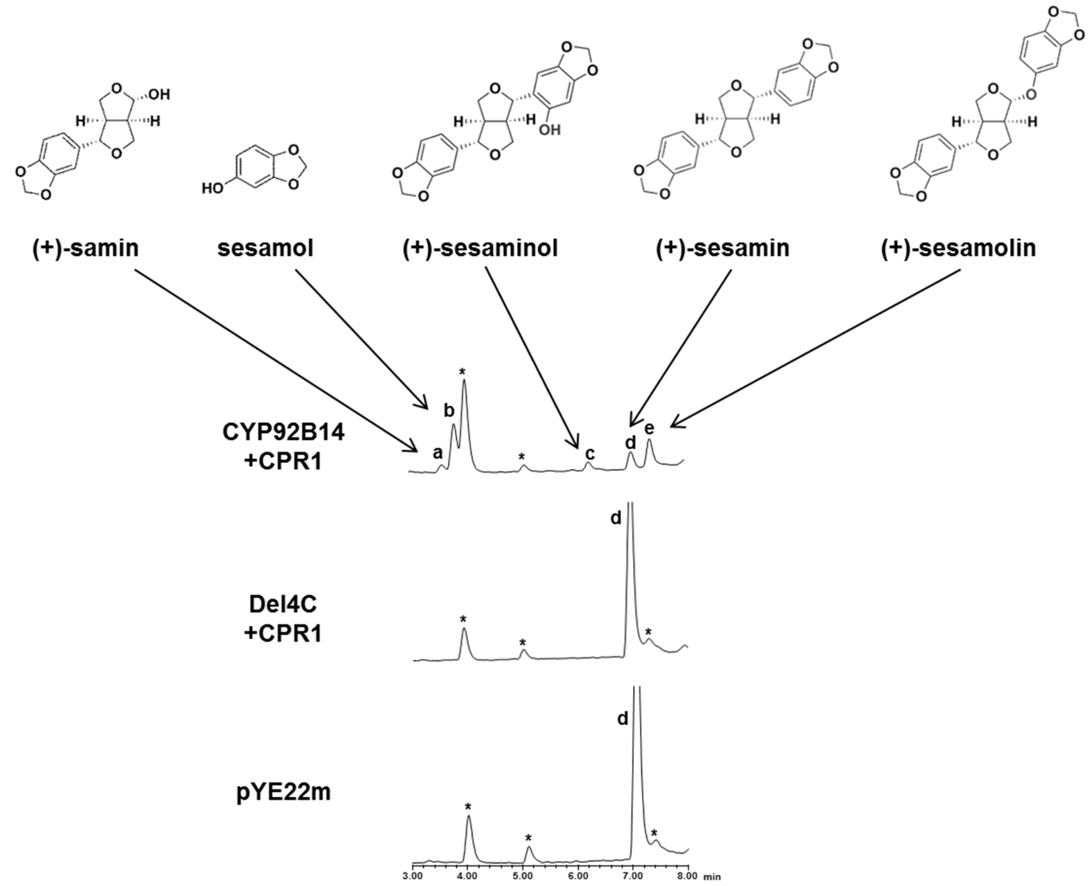

Supplementary Figure 10: Oxygenation of (+)-sesamin is associated with the production of sesamol and (+)-samin. Yeast cell lines expressing either *CYP92B14* and *CPR1* or *Del4C* and *CPR1* were subjected to bioassays using (+)-sesamin as a substrate. Yeast cells harboring the empty vector pYE22m were used as negative control. Assay products after 48 hr incubation were separated by HPLC (Shimadzu) and detected using a fluorescence detector at Ex/Em=280/340 nm. Note that sesamol and (+)-samin were detected only from yeast cells expressing *CYP92B14* and *CPR1*. a: sesamol, b: (+)-samin, c: (+)-sesaminol, d: (+)-sesamin, e: (+)-sesamolin, *: unknown peaks.

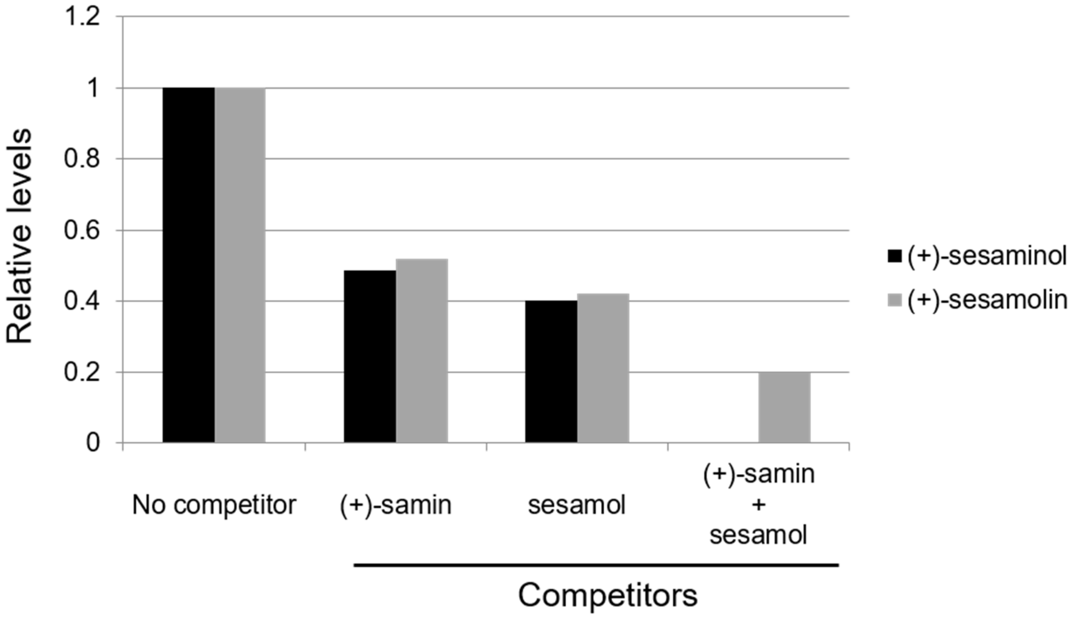

Supplementary Figure 11: (+)-samin and sesamol inhibits CYP92B14-mediated biosynthesis of (+)-sesamolin and (+)-sesaminol. Either or both of 100 μM (+)-samin and 100 μM sesamol was supplemented to the yeast cultures expressing CYP92B14 and CPR1 with 100 μM (+)-sesamin as a substrate. The reaction was analyzed by HPLC after 48 hr of incubation, and the levels of generated (+)-sesamolin and (+)-sesaminol were shown as values relative to those without competitors.

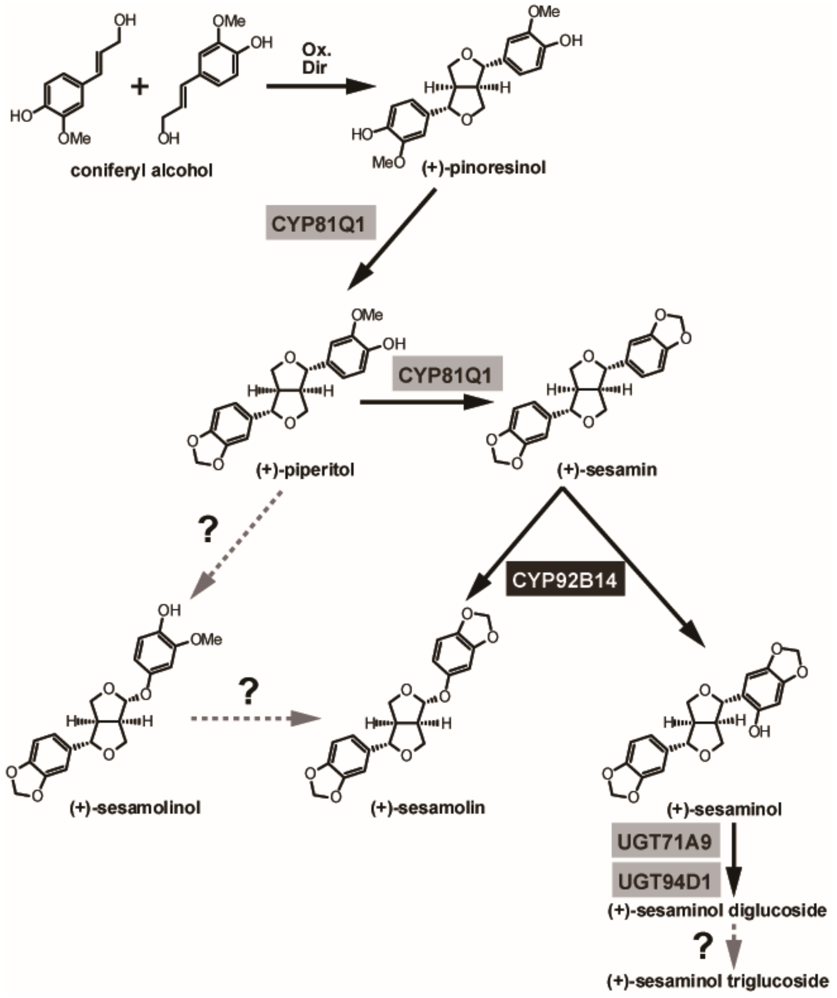

Supplementary Figure 12: Biosynthesis of (+)-sesamolin and (+)-sesaminol from (+)-sesamin by CYP92B14. Proposed biosynthetic pathway for (+)-sesamolin and (+)-sesaminol in *S. indicum*. Solid black arrows: biochemically characterized reactions, dashed grey arrows: unknown reactions, Black rectangule; the enzyme characterized in this study, Grey rectangules: known enzymes, Dir: dirigent protein, Ox.: oxidant.

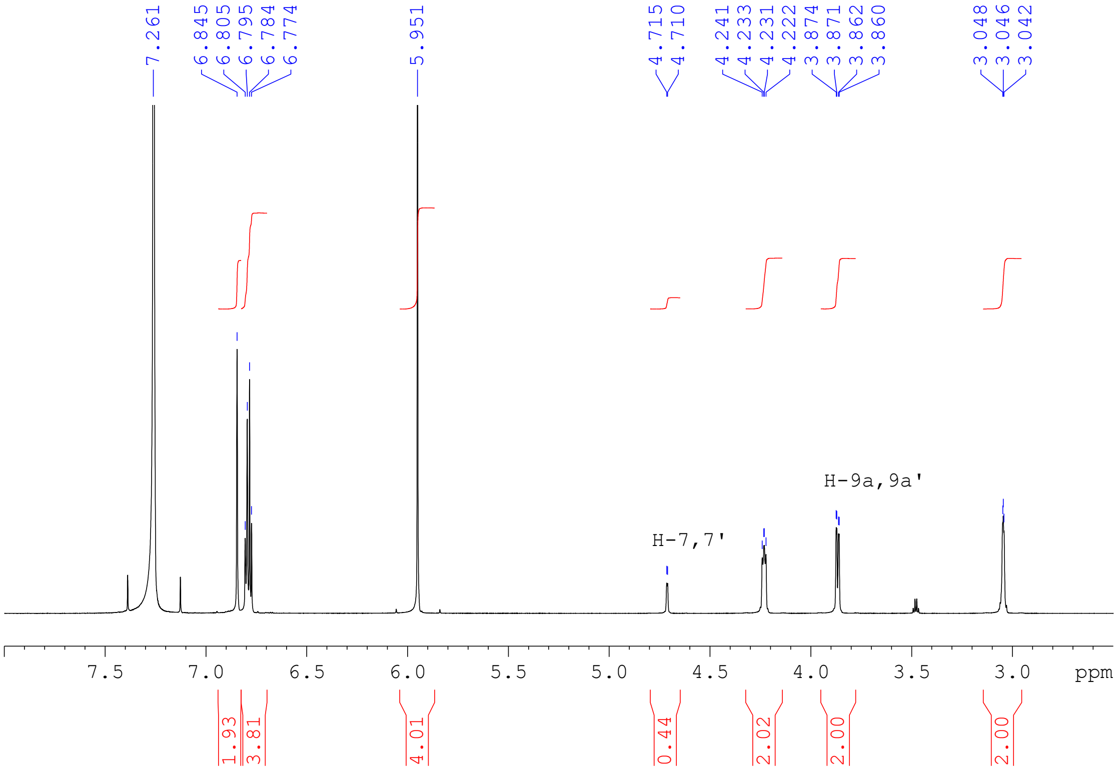

Supplementary Figure 13: ^{1}H NMR data of compound 1

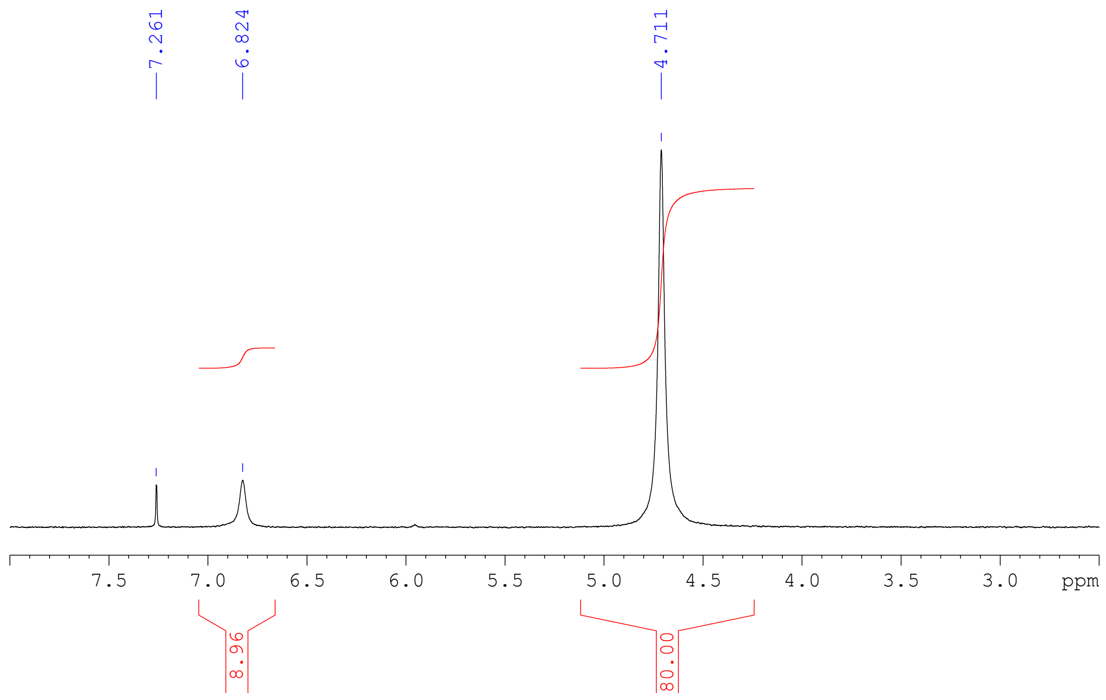

Supplementary Figure 14: ^{2}H NMR data of compound 1

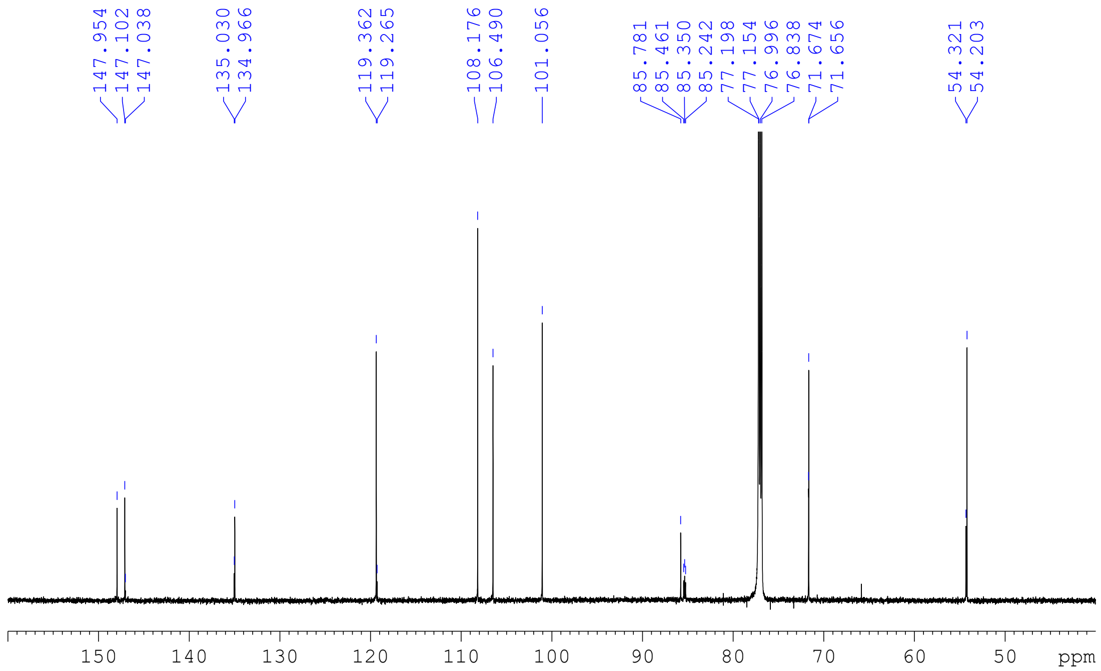

Supplementary Figure 15: ^{13}C NMR data of compound 1

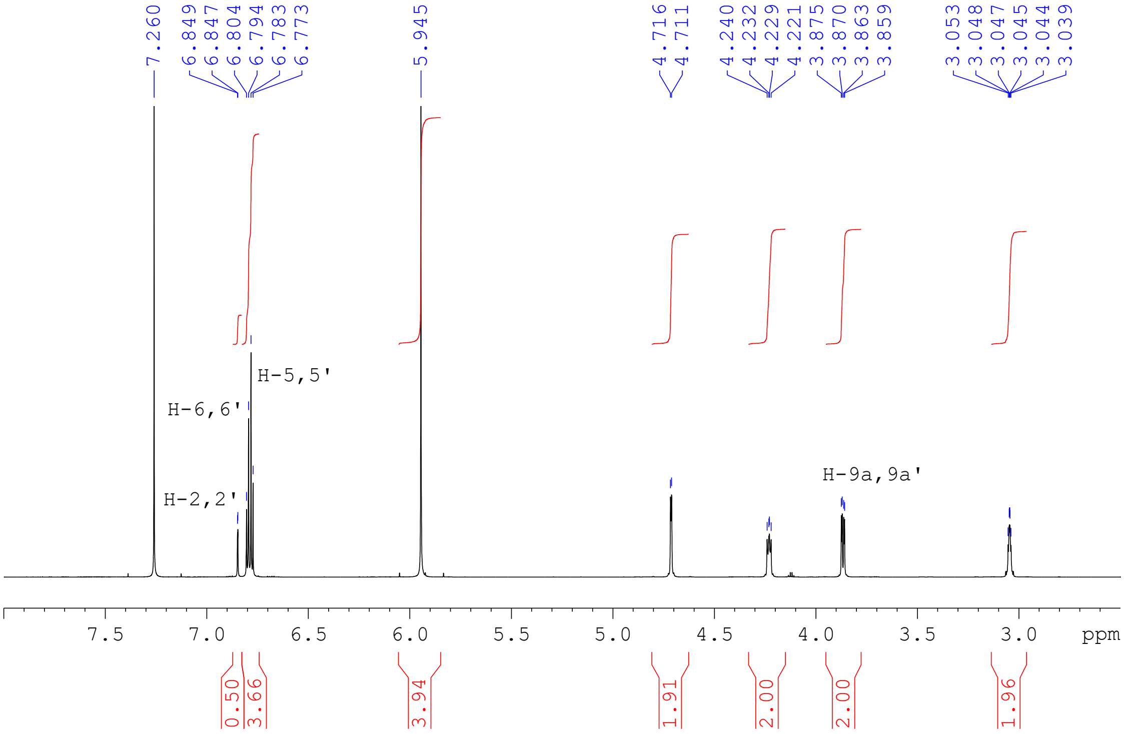

Supplementary Figure 16: ^{1}H NMR data of compound 2

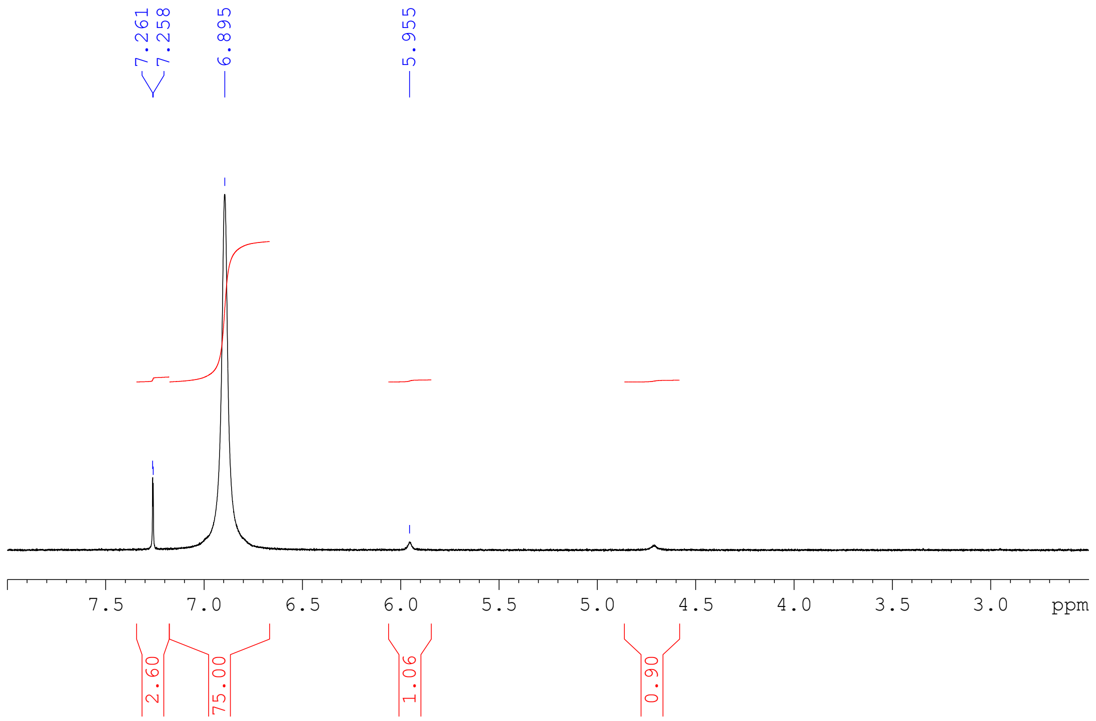

Supplementary Figure 17: ^{2}H NMR data of compound 2

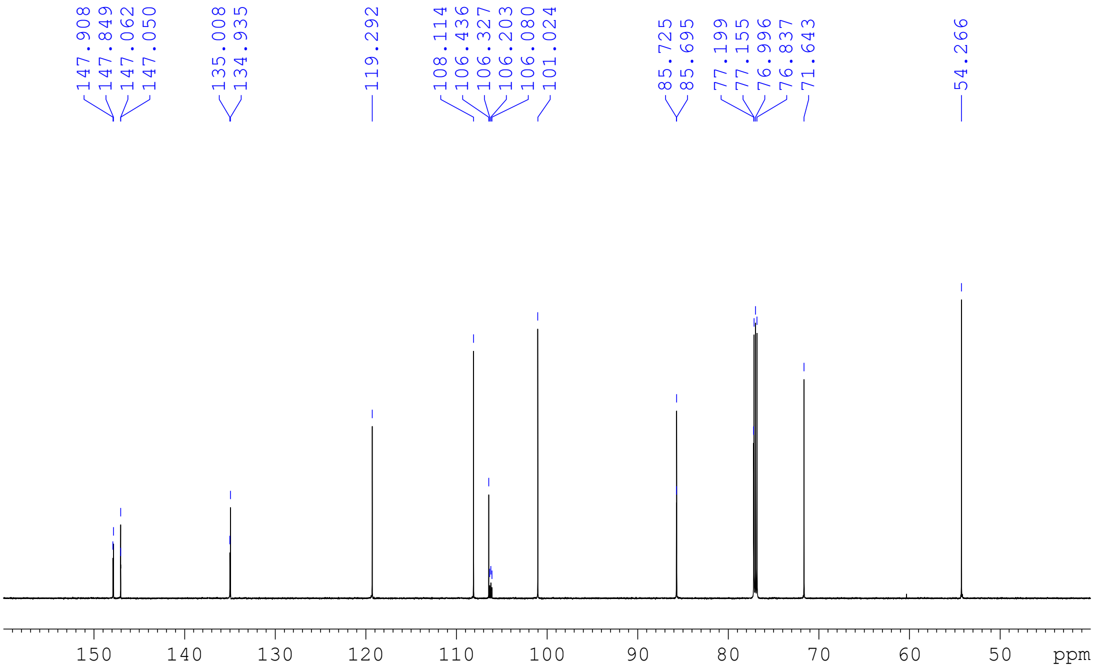

Supplementary Figure 18: ^{13}C NMR data of compound 2

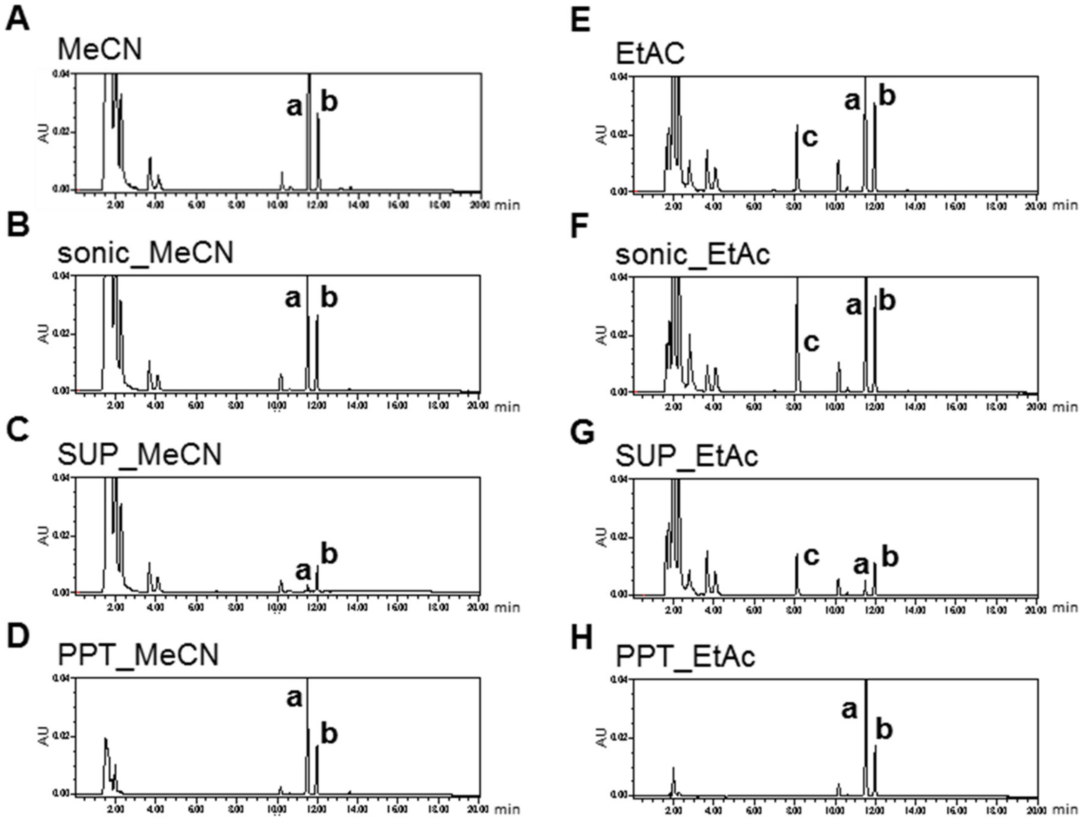

Supplementary Figure 19: The extraction efficiency of lignans from yeast cultures by simple addition of acetonitrile is equivalent to that obtained by ethyl acetate extraction. HPLC analysis was conducted as described in Materials and Methods. **a**: (+)-sesamin, **b**: (+)-sesamolin, **c**: unknown yeast metabolite. (A) MeCN: add an equal volume of acetonitrile. (B) sonic_MeCN: sonicate and add an equal volume of acetonitrile. (C) SUP_MeCN: centrifuge and add an equal volume of acetonitrile to supernatant (SUP). (D) PPT_MeCN: centrifuge, resuspend the pellet (PPT) with an equal volume of dH_2O and add an equal volume of acetonitrile to PPT. (E) EtAc: add an equal volume of ethyl acetate (EtAc) and extract. (F) sonic_EtAc: sonicate, add an equal volume of EtAc and extract. (G) SUP_EtAc: centrifuge, add an equal volume of EtAc to SUP and extract with EtAc. (H) PPT_EtAc: centrifuge, resuspend in an equal volume of dH_2O, and add an equal volume of EtAc to PPT and extract.

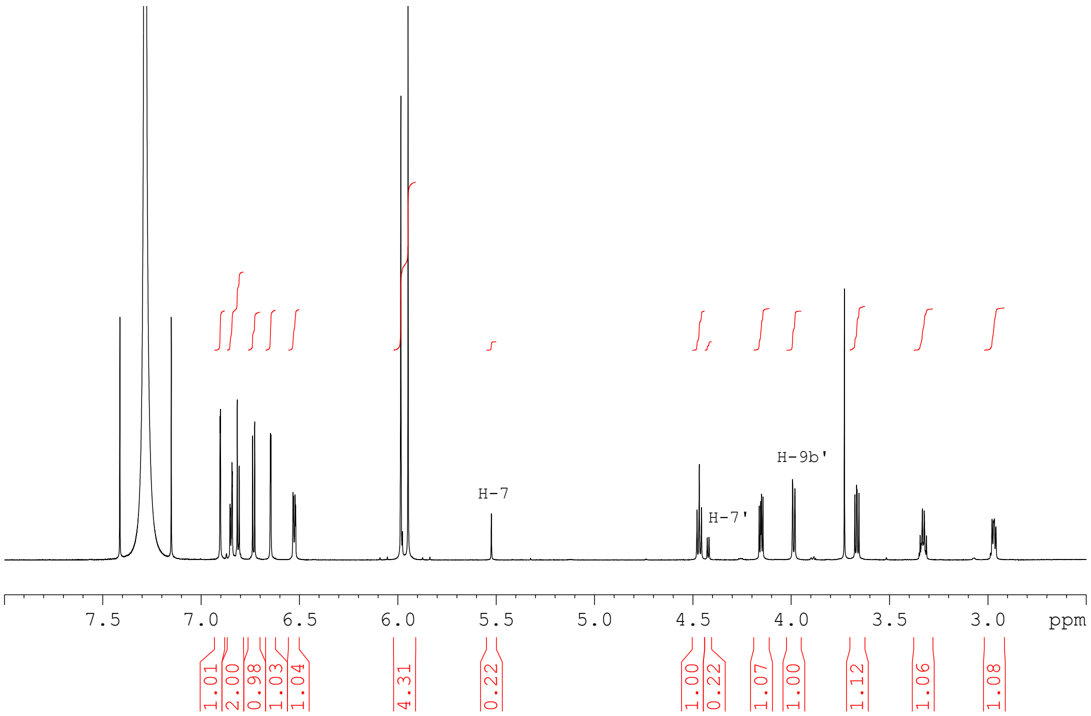

Supplementary Figure 20: ^{1}H NMR data of (+)-sesamolin, enzymatically derived from compound 1

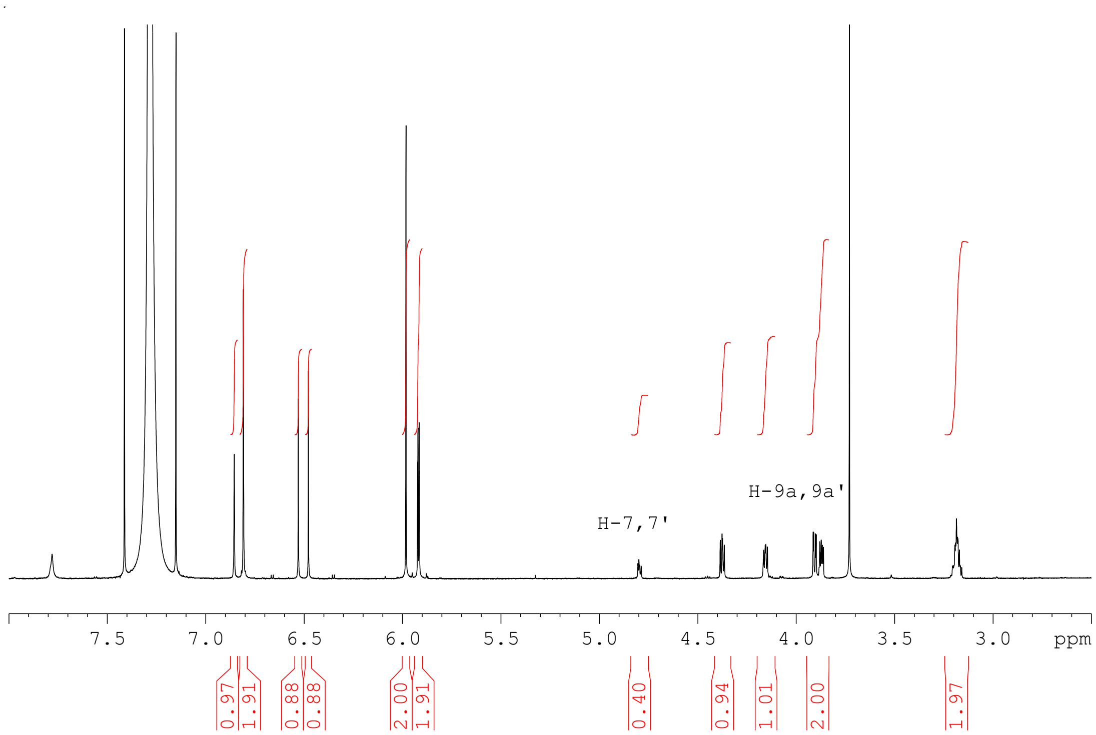

Supplementary Figure 21: ^{1}H NMR data of (+)-sesaminol, enzymatically derived from compound 1

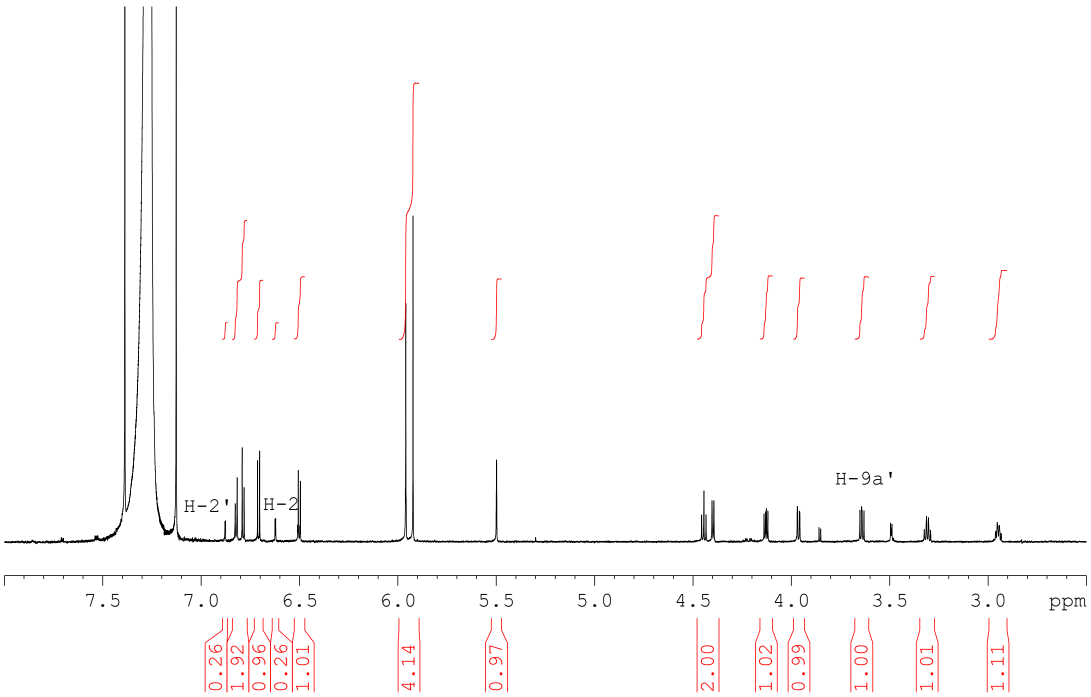

Supplementary Figure 22: ^{1}H NMR data of (+)-sesamolin, enzymatically derived from compound 2

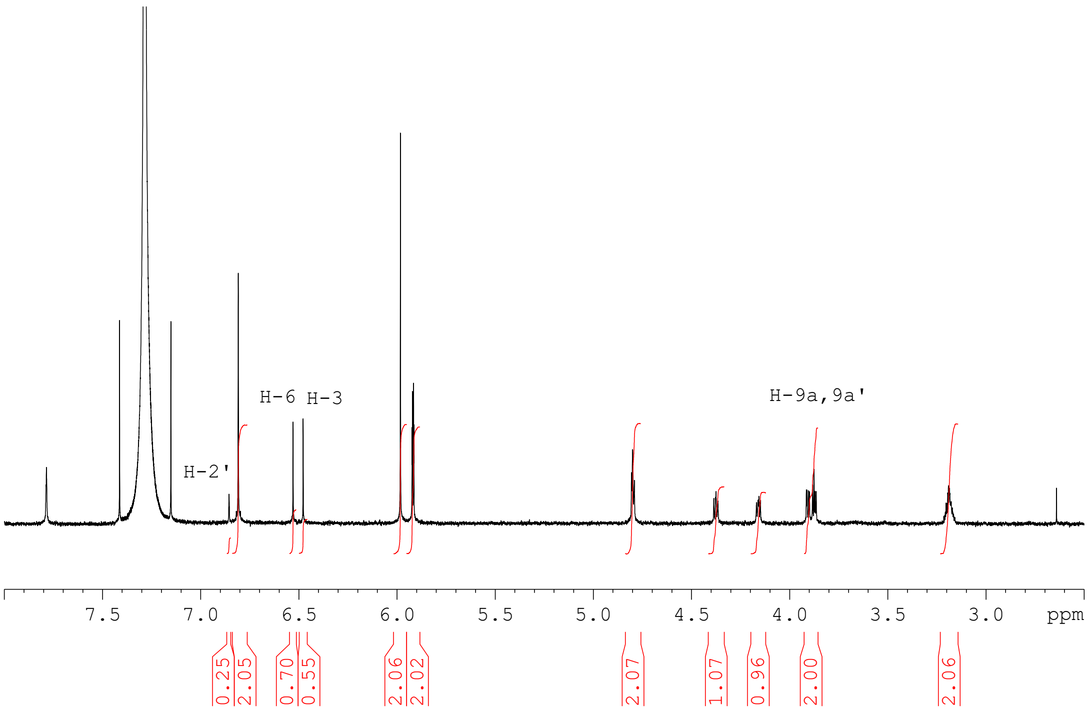

Supplementary Figure 23: ^{1}H NMR data of (+)-sesaminol, enzymatically derived from compound 2

Supplementary Tables:

Supplementary Table 1: Lignan content of seeds from sesame strains and their F1 plants.

	Lignan contents (mg/g seed)	
strain/generation	sesamin	sesamolin
#4294	4.0	0.0
ITCFA2002	9.2	2.0
#4294 x ITCFA2002/F_1	6.9±0.6	1.5±0.1

Supplementary Table 2: Inheritance of the sesamolin-deficient trait in F_6 RILs resulting from the cross between #4294 and ITCFA2002.

generation	sesamolin-accumulating	sesamolin-deficient	χ^2 (ratio)	*P*-value
RILs (F_6)	79	81	0.025 (1:1)	$0.8<P<0.9$

Supplementary Table 3: List of oligonucleotides used for cloning and qPCR analyses.

oligo name	oligo sequence
SiCYP92B14-cloning-FW	5'-TCTTCACTGTTGATAGGTACCATGCCGCTGATGGAGAACTCC-3'
SiCYP92B14-cloning-RV	5'-GATTCAGAATTGTCGACTCAGTAAAGATGGTCAGGAAGTGCA-3'
SiCYP92B14-L1-pYE-FW	5'-CAGAATTCGAGCTCGGTACCATGCCGCTGATGGAGAACTCC-3'
SalI-SiCYP75B-L1-Low-pYE-RV	5'-GTTCAACCAAGTCGACTCAGGAAAGTGCAGGATTCATAATA-3'
Si CPR1-pJH-FW	5'-AGACTGAGCTCGTTTAAACATGGAACCCACATCGGAAAAG-3'
Si CPR1-pJH-RV	5'-TATATTAATCGGATCCTCACCACACATCACGCAAGTAC-3'
Si 18SrRNA-qPCR-FW	5'-CGTCCCTGCCCTTTGTACAC-3'
Si 18SrRNA-qPCR-RV	5'-CGAACACTTCACCGGACCAT-3'
Si CYP81Q1-qPCR-FW	5'-AAGTTGTTGCCGTTCGGAATG-3'
Si CYP81Q1-qPCR-RV	5'-CGTTCTCTCCCAGTCGAAACA-3'
Si CYP92B14-qPCR-FW	5'-TCGATTACTCAGACGTGACATGG-3'
Si CYP92B14-qPCR-RV	5'-GGCTTTCCTGATAGGGAGTGTAG-3'
Si CPR1-qPCR-FW	5'-ATGCTTCTGACCCCAGTGAAG-3'
Si CPR1-qPCR-RV	5'-AATTGGAGGCTTGGCTGATGA-3'

Supplementary Methods:

cDNA fragments of CYP92B14 full-length ORF, Del4C and CPR1 were amplified with primer sets SiCYP92B14-cloning-FW and SiCYP92B14-cloning-RV, SiCYP92B14-L1-pYE-FW and SalI-SiCYP75B-L1-Low-pYE-RV, and Si CPR1-pJH-FW and Si CPR1-pJH-RV, respectively. The amplified fragments of CYP92B14 full-length ORF and Del4C were subcloned to pYE22m[12] using *Kpn*I and *Sal*I sites. The amplified fragment of CPR1 was subcloned to pJHXSBP-His[59] using *Pme*I and *Bam*HI sites. cDNA fragment of CYP81Q1 in pENTR-D-TOPO[39] was subcloned to pYES2-DEST52 (Thermo Fisher Scientific) according to manufacturer's instruction. Sequences that correspond to coding regions within the cloning primers are underlined.

Supplementary References:

55. Wang, L. *et al.* Variation of sesamin and sesamolin contents in sesame cultivars from China. *Pakistan J. Bot.* **45,** 177–182 (2013).

56. Yasumoto, S. & Katsuta, M. Breeding a high-lignan-content sesame cultivar in the prospect of promoting metabolic functionality. *Japan Agric. Res. Q.* **40,** 123–129 (2006).

57. Sato, F. & Kumagai, H. Microbial production of isoquinoline alkaloids as plant secondary metabolites based on metabolic engineering research. *Proc. Japan Acad. Ser. B* **89,** 165–182 (2013).

58. Vanholme, R., Demedts, B., Morreel, K., Ralph, J. & Boerjan, W. Lignin biosynthesis and structure. *Plant Physiol.* **153,** 895–905 (2010).

59. Hatanaka, H., Omura, F., Kodama, Y. & Ashikari, T. Gly-46 and His-50 of yeast maltose transporter Mal21p are essential for its resistance against glucose-induced degradation. *J. Biol. Chem.* **284,** 15448-15457 (2009).
